# Analysis of the Molecular Mechanisms of the Effects of *Prunella vulgaris* against Subacute Thyroiditis Based on Network Pharmacology

**DOI:** 10.1155/2020/9810709

**Published:** 2020-11-10

**Authors:** Xin Shen, Rui Yang, Jianpeng An, Xia Zhong

**Affiliations:** ^1^First Clinical College, Shandong University of Traditional Chinese Medicine, Jinan 250355, China; ^2^Shandong Provincial Hospital Affiliated to Shandong First Medical University, Jinan 250021, China; ^3^Neck-Shoulder and Lumbocrural Pain Hospital of Shandong First Medical University, Jinan 250000, China; ^4^Department of General Practice, Shandong Provincial Hospital Affiliated to Shandong First Medical University, Jinan 250021, China

## Abstract

*Prunella vulgaris* (PV) has a long history of application in traditional Chinese and Western medicine as a remedy for the treatment of subacute thyroiditis (SAT). This study applied network pharmacology to elucidate the mechanism of the effects of PV against SAT. Components of the potential therapeutic targets of PV and SAT-related targets were retrieved from databases. To construct a protein-protein interaction (PPI) network, the intersection of SAT-related targets and PV-related targets was input into the STRING platform. Gene ontology (GO) analysis and KEGG pathway enrichment analysis were carried out using the DAVID database. Networks were constructed by Cytoscape for visualization. The results showed that a total of 11 compounds were identified according to the pharmacokinetic parameters of ADME. A total of 126 PV-related targets and 2207 SAT-related targets were collected, and 83 overlapping targets were subsequently obtained. The results of the KEGG pathway and compound-target-pathway (C-T-P) network analysis suggested that the anti-SAT effect of PV mainly occurs through quercetin, luteolin, kaempferol, and beta-sitosterol and is most closely associated with their regulation of inflammation and apoptosis by targeting the PIK3CG, MAPK1, MAPK14, TNF, and PTGS2 proteins and the PI3K-Akt and TNF signaling pathways. The study demonstrated that quercetin, luteolin, kaempferol, and beta-sitosterol in PV may play a major role in the treatment of SAT, which was associated with the regulation of inflammation and apoptosis, by targeting the PI3K-Akt and TNF signaling pathways.

## 1. Introduction

Subacute thyroiditis (SAT), which is also called subacute granulomatous thyroiditis, de Quervain's thyroiditis, or giant-cell thyroiditis, is the most common cause of thyroid pain. SAT is a self-limited inflammatory thyroid disease possibly related to viral infection that usually presents as a prodrome of low-grade fever, fatigue, goitre, and pharyngitis symptoms [1]. There is no definitive cure for painful SAT, but there are effective treatments that will relieve the symptoms and allow the disease to run its course in an asymptomatic fashion, including nonsteroidal anti-inflammatory agents (NSAIDs) and glucocorticoids. Unfortunately, approximately 5% to 15% of patients develop permanent hypothyroidism after recovering full thyroid function within 12 months [2, 3] and 1% to 4% relapse after a year. [4].

*Prunella vulgaris* (PV) has the functions of clearing fire and dispersing knots and swelling, and it has been confirmed that PV suppresses inflammation via several signal transduction pathways [5]. In recent years, PV and its preparations (such as PV oral liquid) have played an important role in the treatment of SAT. PV is recommended in the treatment of SAT for qi stagnation and phlegm-blocking type and the syndrome of deficiency of both qi and yin according to Chinese Medicine Diagnosis and Treatment Scheme of Gall Pain (SAT) by the Chinese State Administration of Traditional Chinese Medicine in 2017 (trial version). According to the expert consensus statement on the treatment of goitre/nodular thyroid disease with PV in clinical practice according to the Chinese Experts of Clinical Application of PV, the use of PV alone to treat SAT with one course each month for 2-3 courses is recommended (recommended classification: B). PV combined with chemical-based medicine for 4 weeks as a course for 1-2 courses is recommended (recommended classification: B) [6]. Initial treatment combined with PV reduced prednisolone consumption for patients with SAT [7]. However, the molecular mechanisms of the effects of PV against SAT are still unclear.

Network pharmacology, which elucidates the synergistic effects and the underlying mechanism of multiple components and multitargets, has been proven to be a powerful tool for the exploration of Traditional Chinese Medicine [8]. In recent years, network pharmacology has been widely used to investigate the interactions of active ingredients, relevant targets, and molecular mechanisms in TCM [9]. Therefore, the present study was based on network pharmacology to elucidate the mechanism of the effects of PV against SAT.

**Figure 1:**
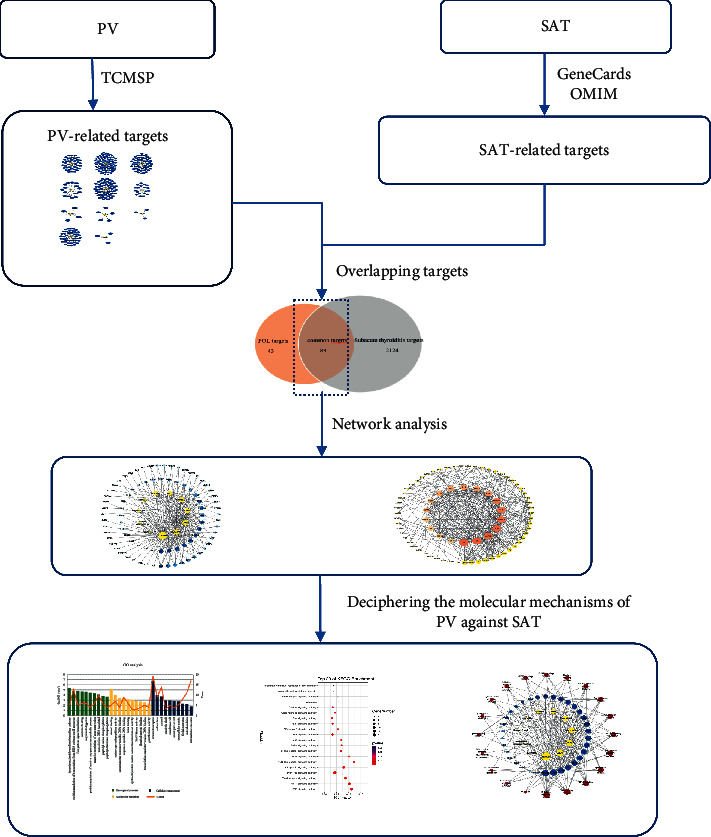
The graphical abstract of this study. PV, *Prunella vulgaris*; SAT, subacute thyroiditis.

In the present study, network pharmacology analysis, including the identification of active ingredients, prediction of ingredient-related targets and SAT-related targets, construction of a protein-protein interaction (PPI) network, and gene ontology (GO) and KEGG pathway analysis, was subsequently performed to clarify the biological processes related to the target protein involved in the PV-mediated treatment of SAT. The workflow is as follows (Figure 1).

## 2. Materials and Methods

### 2.1. Identification of Components and Targets of PV

The PV-related targets were retrieved from the Pharmacology Database and Analysis Platform (TCMSP, http://lsp.nwsuaf.edu.cn/tcmsp.php) based on the term “Xiaokucao” to search for the effective components of PV. Then, according to the pharmacokinetic parameters of ADME, components meeting the criteria of both an OB (Oral Bioavailability) ≥30% and a DL (Drug-Likeness) ≥0.18 were defined as active ingredients [10]. OB represents the pharmacological percentage of an oral drug entering the systemic circulation, and a high OB is usually defined as the key index to determine the bioactive molecules in therapeutic drugs. DL is a qualitative concept used to estimate the drug properties of compounds, which is helpful to optimize the pharmacokinetics and drug properties, such as solubility and chemical stability [11]. Subsequently, candidate compounds were input into TCMSP to search for related protein targets.

### 2.2. Prediction of Disease-Related Genes

Screening of SAT-related genes from the GeneCards database (https://www.genecards.org/) and OMIM database (http://www.omim.org/) was performed. The keywords, “subacute thyroiditis,” “subacute granulomatous thyroiditis,” “de Quervain's thyroiditis,” and “giant-cell thyroiditis,” were used, and the targets were the human genes identified in this study. The intersection of compound targets and disease targets is considered to be therapeutic targets.

### 2.3. Network Construction

Compound-target (C-T), compound-overlapping target (C-OT), and compound-target-pathway (C-T-P) networks were constructed by Cytoscape 3.7.2. The topological parameters of the nodes were calculated by a “network analyzer,” including the degree centrality (DC) and betweenness centrality (BC). Degree refers to the number of adjacent proteins. BC is another standard measure of node centrality in a network, which measures the total number of nonredundant shortest paths going through a certain node or edge; both parameters are considered to be great predictors of essentiality in the interaction network [12, 13]. In the network, nodes represent compounds, targets, and pathways, and edges represent the relationship between compounds and targets and between targets and pathways.

To clarify the direct or indirect regulatory relationship between therapeutic targets, we used the STRING platform (https://string-db.org/) to construct a PPI network, with the species limited to “*Homo sapiens*” and high confidence (minimum required interaction score ≥0.7). Then, the network data were downloaded and input into Cytoscape 3.7.2, and the topological parameters of the nodes were calculated by a “network analyzer.” The nodes with DC scores and BC scores greater than 2 times the median were considered as important nodes in the network, namely, as hub targets [14]. In the network, nodes represent targets, and edges represent target interactions.

### 2.4. Pathway and Functional Enrichment Analysis

To clarify the role of therapeutic target proteins in gene function and signaling pathways, the therapeutic target proteins were submitted to the DAVID database for GO function enrichment analysis and KEGG pathway enrichment analysis. *P* < 0.05 was considered significant.

## 3. Results

### 3.1. Components and Targets of P

**Figure 2:**
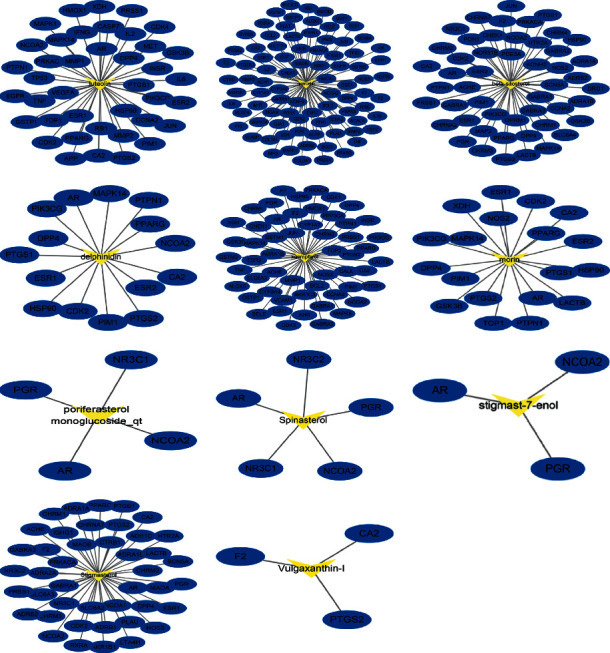
The C-T network of PV. The yellow nodes represent active compounds, and the blue nodes represent compounds targets of each compound. C-T network: compound-target network; PV, *Prunella vulgaris.*

**Figure 3:**
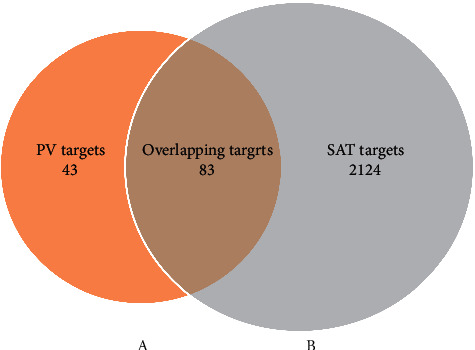
Overlapping targets between 126 PV-related targets (A) and 2207 SAT-related targets (B). PV, *Prunella vulgaris*; SAT, subacute thyroiditis.

**Figure 4:**
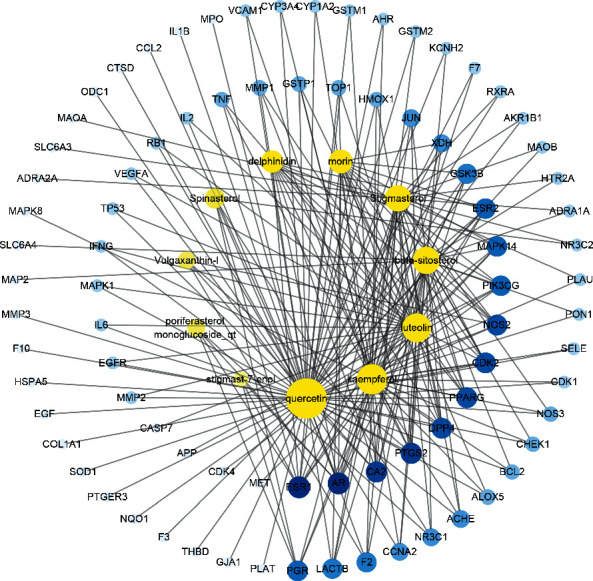
The C-OT network of PV in treating SAT. The yellow nodes represent active compounds, and the blue nodes represent therapeutic targets. Nodes size and color depth are proportional to their degree. C-OT network: compound-overlapping target network. PV, *Prunella vulgaris*; SAT, subacute thyroiditis.

A total of 60 components and 335 targets were retrieved from the TCMSP database. Finally, 11 components were selected as active ingredients based on the criteria of OB ≥ 30% and DL ≥ 0.18 (Table 1), and 126 targets remained after removing duplicate targets, as shown in the C-T network (Figure 2, Table S1). In addition, 3079 SAT-related targets were selected from the database, and 2207 targets remained after deleting duplicate targets (Table S2). After determination of the intersection of the drug target and disease target, 83 targets were considered to be potential therapeutic targets for treatment of SAT for constructing the C-OT network, which included 94 nodes (11 compounds and 83 targets) and 231 edges (Figures 3 and 4, Table 1). From the perspective of active ingredients, each ingredient was connected with multiple targets. Quercetin (degree = 68) interacted with 68 targets and exhibited the highest correlation with disease targets, and the rest of the active ingredients included kaempferol (degree = 37), luteolin (degree = 32), beta-sitosterol (degree = 27), and stigmasterol (degree = 24). From the perspective of targets, most targets were connected with more than 2 ingredients, and the top 5 targets based on degree were AR (degree = 10), ESR1 (degree = 10), PTGS2 (degree = 8), CA2 (degree = 8), and NOS2 (degree = 7). Moreover, to exhibit biological properties, components and metabolites need to be available in the target tissue. Therefore, the biological properties of components also depend on their absorption in the gut and the OB. The top 3 components ranked according to the OB were vulgaxanthin-I (OB = 56.14%), quercetin (OB = 46.43%), and morin (OB = 46.23%). The abovementioned data show that PV plays a complex role in multiple components and targets in the whole biological function system.

### 3.2. PPI Network and Hub Target Screening

**Figure 5:**
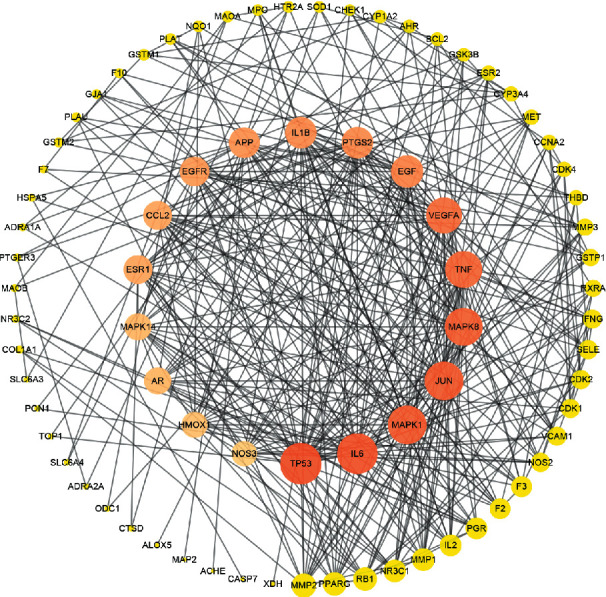
The PPI network of overlapping targets. The colors of the nodes are illustrated from orange to yellow in descending order of degree values. Nodes size is proportional to their degree. PPI network: protein-protein interaction network.

To obtain information on the predicted interaction, we uploaded the 83 therapeutic targets to the STRING platform, and the results were imported into Cytoscape 3.7.2 to visualize and analyze the interaction network (Figure 5). There were 77 nodes and 383 edges in the network. According to the topological parameters of the network, the median DC was 7, and the median BC was 0.00287026. Finally, 18 targets were selected as hub targets based on DC and BC values that were 2-fold higher than the median, accounting for 21.69% of the total targets. As shown in Table 2, TP53, which showed the highest degree, can act on 33 targets, and the rest of the genes ranked according to the degree value were IL6, JUN, MAPK1, MAPK8, TNF, VEGFA, EGF, IL-1*β*, PTGS2, APP, EGFR, ESR1, CCL2, MAPK14, AR, HMOX1, and NOS3.

### 3.3. GO Analysis of Therapeutic Targets

**Figure 6:**
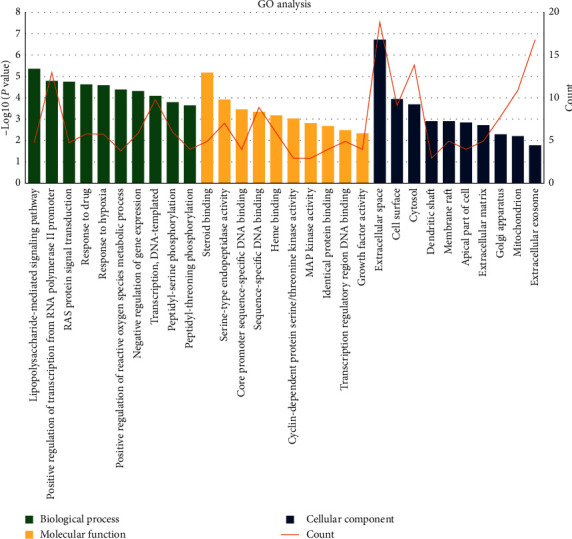
GO enrichment analysis of 83 therapeutic targets. GO, gene ontology.

To elaborate the biological functions of therapeutic targets, we submitted 83 therapeutic targets to the DAVID platform. As a result, a total of 168 GO terms were selected, including 107 biological process (BP) terms (Table S3), 36 molecular function (MF) terms (Table S4) and 25 cellular component (CC) terms (Table S5). We screened the top 10 significantly enriched terms in the BP, MF, and CC categories, as shown in Figure 6. The results indicated that therapeutic targets were related to the lipopolysaccharide-mediated signaling pathway, Ras protein, response to hypoxia, reactive oxygen species metabolic process in BP, steroid binding, serine-type endopeptidase activity, core promoter sequence-specific DNA binding, sequence-specific DNA binding, heme binding, cyclin-dependent protein serine/threonine kinase activity in MF, and the extracellular space, cell surface, cytosol, dendritic shaft, and membrane raft in CC.

### 3.4. KEGG Pathway Analysis and C-T-P Network Construction

**Figure 7:**
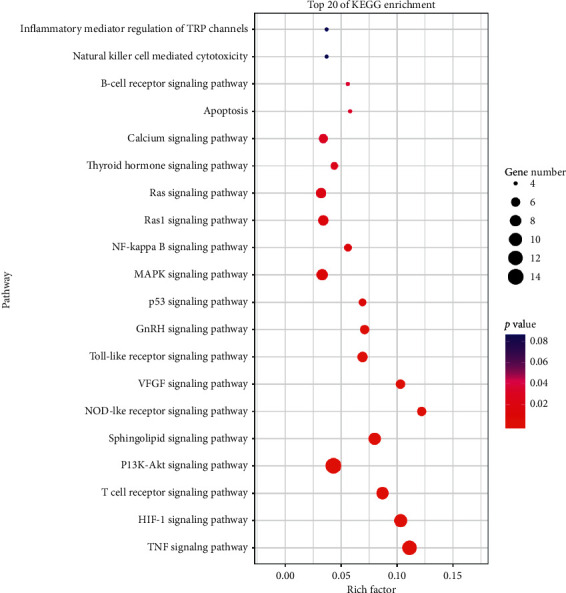
KEGG analysis of 83 therapeutic targets.

A total of 90 KEGG pathways were retrieved (Table S6), and we screened the top 20 pathways after removing uncorrelated pathways such as “bladder cancer” and “Chagas disease” according to a *P* value < 0.05 (Figure 7). The result indicated that the targets were most closely related to the TNF signaling pathway, and the other pathways ranked by the *P* value were the HIF-1 signaling pathway, T-cell receptor signaling pathway, PI3K-Akt signaling pathway, sphingolipid signaling pathway, NOD-like receptor signaling pathway, VEGF signaling pathway, and toll-like receptor signaling pathway, which were mainly related to inflammation, hypoxia, immunity, and angiogenesis.

**Figure 8:**
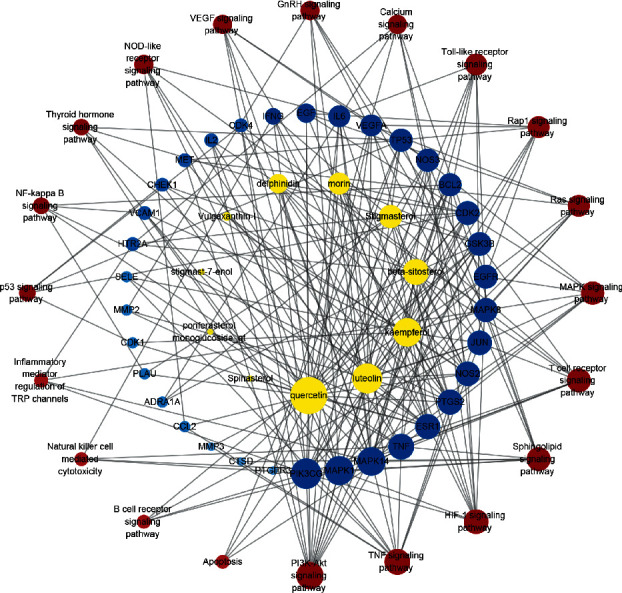
The C-T-P network constructed by Cytoscape. The yellow nodes represent active compounds, the blue nodes represent targets, and red nodes represent pathways. Nodes size and color depth are proportional to their degree. C-T-P network: compound-target-pathway network.

A C-T-P network was constructed based on the top 20 pathways and involved targets and the corresponding compounds by Cytoscape 3.7.2 to further illustrate the molecular biological process of PV treatment of SAT (Figure 8). A total of 65 nodes (11 compounds, 34 targets, and 20 pathways) and 239 edges were obtained. The topological parameters of the C-T-P network, including the DC and BC, were analyzed by a network analyzer in Cytoscape 3.7.2 (Table 3). First, from the perspective of the compound, the average DC and BC values were 9.18 and 0.049, respectively, and 4 compounds with DC and BC values higher than the average values were quercetin (DC = 29, BC = 0.28053366), luteolin (DC = 18, BC = 0.09443417), kaempferol (DC = 16, BC = 0.07144764), and beta-sitosterol (DC = 12, BC = 0.05629246). Then, from the perspective of targets, the average DC and BC values were determined to be 7.03 and 0.021, respectively, and there were 15 targets with DC and BC values higher than the average values: PIK3CG (DC = 19, BC = 0.10183777), MAPK1 (DC = 17, BC = 0.06698717), MAPK14, (DC = 16, BC = 0.06811842), TNF (DC = 12, BC = 0.03951742), PTGS2 (DC = 11, BC = 0.0446649), ESR1 (DC = 11, BC = 0.10775069), JUN (DC = 10, BC = 0.02513484), NOS2 (DC = 10, BC = 0.0391081), MAPK8 (DC = 9, BC = 0.01600203), EGFR (DC = 9, BC = 0.02564519), GSK3B (DC = 9, BC = 0.01963646), CDK2 (DC = 9, BC = 0.02597067), BCL2 (DC = 8, BC = 0.01961612), NOS3 (DC = 8, BC = 0.02216569), and TP53 (DC = 8, BC = 0.02125164). Next, from the perspective of pathways, the average DC and BC values were 6.9 and 0.011, respectively, which corresponded to the PI3K-Akt signaling pathway (DC = 14, BC = 0.04279241), TNF signaling pathway (DC = 12, BC = 0.03360015), HIF-1 signaling pathway (DC = 10, BC = 0.01836123), T-cell receptor signaling pathway (DC = 9, BC = 0.01596886), and sphingolipid signaling pathway (DC = 9, BC = 0.01803947).

## 4. Discussion

In the present study, we studied the molecular mechanism of the effects of PV on the treatment of SAT with network pharmacology. A total of 11 components were eventually retrieved, which mainly included flavones and nonsteroids according to the criteria of OB ≥ 30% and DL ≥ 0.18. Quercetin, kaempferol, and luteolin, which are plant flavonoids, exhibited high topological values in the C-OT network, suggesting that these three components play an important role in treating SAT. Quercetin, exhibiting the highest degree value and a relatively high OB, has been demonstrated to improve thyroid function in vivo. It has been demonstrated that quercetin has a therapeutic effect on hyperthyroidism and could be used to protect against experimental hyperthyroidism-induced liver damage via the MAPK/Nrf2 pathway [15]. Additionally, it can alleviate the abnormal haemostasis caused by methimazole-induced hypothyroidism in rats by modulating the hydrolysis of adenine nucleotides and nucleosides and consequently reducing platelet aggregation [16]. Kaempferol acted as a competitive TPO inhibitor regulating the synthesis of thyroid hormone in a previous study. Luteolin has been shown to possess anti-inflammatory activity both in vitro and in vivo [17, 18], and it has potent anti-inflammatory effects on murine experimental autoimmune thyroiditis [19]. In general, each ingredient is connected with multiple targets, and different components derived from different herbs act on common targets. These components of phytomedicines exert therapeutic effects through the synergistic action of several chemical compounds acting at multiple target sites.

Through the topological analysis of the PPI network, we identified 18 hub genes, including TP53, IL6, JUN, MAPK1, MAPK8, TNF, VEGFA, EGF, IL-1*β*, PTGS2, APP, EGFR, ESR1, CCL2, MAPK14, AR, HMOX1, and NOS, which can be regarded as the hub targets of PV in the treatment of SAT. These genes are associated with inflammation, hypoxia, apoptotic processes, immune responses, oxidative stress, angiogenesis, and other processes. For example, IL6, together with tumor necrosis factor-alpha (TNF-*α*), and IL-1*β* are proinflammatory cytokines that serve as biomarkers for monitoring disease activity and predicting disease severity [20, 21]. Jun family members (c-jun, JunB, and JunD) and one Fos family member (c-fos, FosL1, FosL2, and FosB) participate in the formation of AP-1 complexes. c-jun and c-Fos are target genes for the treatment of inflammation, cancer, and vascular reconstruction and can regulate the expression of many downstream genes, such as CCL2 and IL-1*β*. JunB-independent AP-1 family members mediate the promotion of endothelial proliferation by VEGF, whereas induction of JunB expression primarily mediates VEGF-induced endothelial migration [22]. VEGFA is the most important VEGF, and VEGF is a tyrosine kinase receptor and a key factor for vascular development and formation of new blood vessels (angiogenesis) [23]. EGFR is a 170 kDa transmembrane tyrosine kinase protein, and EGF is one of the ligands of EGFR; EGF can inhibit the uptake and activation of iodine in vivo and in vitro [24], reduce the mRNA and protein expression of TG and TPO, and prevent the release of thyroid hormone to the circulatory system [25]. EGFR triggers downstream signaling pathways such MAPK and PI3K-Akt.

Through the GO enrichment analysis of the therapeutic targets, one interesting phenomenon was observed: the therapeutic effect is mainly observed on the cell surface and in the extracellular space, and it is observed to a lesser degree in the cytosol. This result can be explained by the pathogenesis of SAT. This disease is thought to be caused by a viral infection or a postviral inflammatory process, and double-stranded RNA (dsRNA) formed intracellularly in thyrocytes may be a cause of thyroid dysfunction; therefore, autoimmunity may play a secondary role in pathogenesis [26, 27]. dsRNAs are recognized as pathogen-associated molecular patterns (PAMPs) by TLRs, and the recognition of viral dsRNA induces type I interferon production and activates innate immune responses. [27, 28]. Upon stimulation with TLR by PAMPs, proinflammatory cytokines such as IL-1, TNF-*α*, and IL-6, which modulate thyroid hormonogenesis, were produced [27, 29, 30]. In the pathogenesis process, viral recognition and transmembrane signal transduction occur upstream of the whole signal transduction pathway, which play a decisive role in subsequent events. Components of PV may play an important role at this stage.

By integrating the KEGG pathway analysis with the C-T-P network, we speculate that PV exerts its therapeutic effects against SAT mainly through quercetin, luteolin, kaempferol, and beta-sitosterol and is most closely associated with their regulation of inflammation and apoptosis by targeting the PIK3CG, MAPK1, MAPK14, TNF, and PTGS2 proteins and the PI3K-Akt and TNF signaling pathways, according to the topological parameters. The PI3K/Akt pathway functions in the transmission of cell signaling through transduction systems to the cell nucleus, where it plays an important role in cell growth, proliferation, apoptosis, migration, and differentiation [31]. Activation of PI3K/Akt signaling is believed to be a strategy to increase cell survival and proliferation by viruses to suppress apoptosis [32]. Downregulating the phosphorylation level of PI3K/Akt signaling proteins can promote thyroid epithelial cell (Nthy-ori 3-1) apoptosis [33]. A previous study showed that suppressing the PI3K/Akt/mTOR signaling pathway inhibited autophagy in a model of thyroiditis [34]. TNF-*α* is a multifunctional cytokine with proinflammatory and anti-inflammatory characteristics that plays a critical role in almost every type of inflammatory pathogenesis [35]. It has been demonstrated that flavonoids, including quercetin, kaempferol, and luteolin, possess a benefit in reducing expression of TNF-*α* and other cytokines. Both quercetin and luteolin at 25 *μ*M effectively decreased TNF-*α*, IL-6, IFN-*γ*, and IL-1*β* production in human whole blood incubated with LPS [36]. Moreover, quercetin and luteolin have an effect on the modulation of transcriptional factors; for example, NF-*κ*B and AP-1 are important transcriptional factors involved in the modulation of proinflammatory mediators. Quercetin (100 *μ*M) significantly decreased NF-*κ*B and AP-1 activity in high glucose-induced rat aortic endothelial cells. Luteolin (1–10 *μ*M) significantly inhibited TNF-*α* and IL-6 production and MAPK (JNK and p38) and transcriptional factor (NF-*κ*B and AP-1) activation in SW982 cells [37]. These results prove the accuracy of our network pharmacology analysis.

## 5. Conclusions

In conclusion, a total of 11 components and 83 overlapping targets between subacute SAT-related and PV-related targets, as well as 18 hub genes and 90 pathways, were retrieved by network analysis. The results of the KEGG pathway and C-T-P network analysis suggested that the active components of PV, including quercetin, luteolin, kaempferol, and beta-sitosterol, may play a critical role in the treatment of SAT by targeting the PIK3CG, MAPK1, MAPK14, TNF, and PTGS2 proteins and the PI3K-Akt and TNF signaling pathways. The present study provides evidence to support the further study of PV for the treatment of SAT.

## Figures and Tables

**Table 1:** A total of 11 active ingredients in PV and their degree in the C-OT network.

Number	Compound name	OB (%)	DL	Degree
MOL000098	Quercetin	46.43	0.28	68
MOL000422	Kaempferol	41.88	0.24	37
MOL000006	Luteolin	36.16	0.25	32
MOL000358	Beta-sitosterol	36.91	0.75	27
MOL000449	Stigmasterol	43.83	0.76	24
MOL000737	Morin	46.23	0.27	16
MOL004798	Delphinidin	40.63	0.28	11
MOL004355	Spinasterol	42.98	0.76	5
MOL006767	Vulgaxanthin-I	56.14	0.26	4
MOL006772	Poriferasterol monoglucoside_qt	43.83	0.76	4
MOL006774	Stigmast-7-enol	37.42	0.75	3

**Table 2:** The topological parameters of hub targets.

Number	Node	Degree centrality (DC)	Betweenness centrality (BC)
1	TP53	33	0.146655
2	IL6	32	0.103348
3	JUN	30	0.076404
4	MAPK1	30	0.062201
5	MAPK8	29	0.089256
6	TNF	28	0.057882
7	VEGFA	27	0.065223
8	EGF	23	0.053058
9	IL-1*β*	22	0.009747
10	PTGS2	22	0.044508
11	APP	21	0.191657
12	EGFR	20	0.021937
13	ESR1	19	0.028738
14	CCL2	19	0.013005
15	MAPK14	17	0.004943
16	AR	17	0.026691
17	HMOX1	16	0.01145
18	NOS3	15	0.003683

**Table 3:** The topological parameters of the C-T-P network.

Number	Node	Degree centrality (DC)	Betweenness centrality (BC)
1	Quercetin	29	0.28053366
2	Luteolin	18	0.09443417
3	Kaempferol	16	0.07144764
4	Beta-sitosterol	12	0.05629246
5	Stigmasterol	8	0.02265721
6	Morin	7	0.01050266
7	Delphinidin	6	0.00842733
8	Vulgaxanthin-I	2	1.68*E* − 04
9	Spinasterol	1	0
10	Poriferasterol monoglucoside_qt	1	0
11	Stigmast-7-enol	1	0
12	PIK3CG	19	0.101838
13	MAPK1	17	0.066987
14	MAPK14	16	0.068118
15	TNF	12	0.039517
16	PTGS2	11	0.044665
17	ESR1	11	0.107751
18	JUN	10	0.025135
19	NOS2	10	0.039108
20	MAPK8	9	0.016002
21	EGFR	9	0.025645
22	GSK3B	9	0.019636
23	CDK2	9	0.025971
24	BCL2	8	0.019616
25	NOS3	8	0.022166
26	TP53	8	0.021252
27	IL6	7	0.010758
28	VEGFA	7	0.009984
29	EGF	6	0.007181
30	IFNG	5	0.005949
31	VCAM1	4	0.003979
32	CDK4	4	0.004304
33	IL2	4	0.002602
34	MET	4	0.002161
35	CHEK1	4	0.006204
36	HTR2A	4	0.004661
37	CCL2	3	0.001973
38	SELE	3	0.001084
39	MMP2	3	0.002267
40	CDK1	3	0.003758
41	PLAU	3	0.00391
42	ADRA1A	3	0.001579
43	MMP3	2	4.95*E* − 04
44	CTSD	2	6.58*E* − 04
45	PTGER3	2	0.002331
46	PI3K-Akt signaling pathway	14	0.042792
47	TNF signaling pathway	12	0.0336
48	HIF-1 signaling pathway	10	0.018361
49	T Cell receptor signaling pathway	9	0.015969
50	Sphingolipid signaling pathway	9	0.018039
51	MAPK signaling pathway	8	0.010468
52	Toll-like receptor signaling pathway	7	0.005861
53	Rap1 signaling pathway	7	0.008431
54	Ras signaling pathway	7	0.009325
55	NOD-like receptor signaling pathway	6	0.005763
56	VEGF signaling pathway	6	0.005997
57	GnRH signaling pathway	6	0.006022
58	Calcium signaling pathway	6	0.012494
59	p53 signaling pathway	5	0.004671
60	NF-kappa B signaling pathway	5	0.004721
61	Thyroid hormone signaling pathway	5	0.007399
62	Apoptosis	4	0.002136
63	B-cell receptor signaling pathway	4	0.001427
64	Natural killer cell mediated cytotoxicity	4	0.002105
65	Inflammatory mediator regulation of TRP channels	4	0.006817

## Data Availability

The data used to support the findings of this study are available from the corresponding author upon request.

## Supplementary Materials

Supplementary Materials Table S1: 126 targets of all ingredients. Table S2: 2207 targets of SAT. Table S3: 107 biological process terms associated with therapeutic targets. Table S4: 36 molecular function terms associated with therapeutic targets. Table S5: 25 cellular component terms associated with therapeutic targets. Table S6: 90 KEGG pathways terms associated with therapeutic targets. .
